# Secondhand Smoking and the Risk of Esophageal Squamous Cell Carcinoma in a High Incidence Region, Kashmir, India: A Case-control–observational Study

**DOI:** 10.1097/MD.0000000000002340

**Published:** 2016-01-08

**Authors:** Rumaisa Rafiq, Idrees Ayoub Shah, Gulzar Ahmad Bhat, Mohd Maqbool Lone, Farhad Islami, Paolo Boffetta, Nazir Ahmad Dar

**Affiliations:** From the Department of Biochemistry, University of Kashmir, Hazratbal (RR, IAS, GAB, NAD); Department of Radiation Oncology, SK Institute of Medical Sciences, Soura, Srinagar, JK, India (MML); Surveillance and Health Services Research, American Cancer Society, Atlanta, GA (FI); Digestive Oncology Research Center, Digestive Disease Research Institute, Tehran University of Medical Sciences, Tehran, Iran (FI); and The Tisch Cancer Institute and Institute for Transitional Epidemiology, Mount Sinai School of Medicine, New York, NY (PB).

## Abstract

Studies have associated secondhand smoking (SHS) with cancers of the lung, larynx, and pharynx. Only a few studies have examined the association between SHS and esophageal squamous cell carcinoma (ESCC) and the findings are inconclusive. We aimed to investigate the association between SHS and risk of ESCC in a case-control study in Kashmir, where the incidence of ESCC is high.

We recruited 703 histopathologically confirmed ESCC cases and 1664 hospital-based controls individually matched to the cases for age, sex, and district of residence. Odds ratios (ORs) and 95% confidence intervals (95% CIs) were calculated using conditional logistic regression models.

Among never-tobacco users, the ORs for the association between SHS and ESCC risk were above unity with ever exposure to SHS (OR = 1.32; 95% CI, 0.43–4.02) and exposure to SHS for >14 h/wk (median value) (OR = 2.69; 95% CI, 0.75–20.65). In the analysis of data from all participants, the OR (95% CI) for the association between SHS and ESCC was (OR = 1.02; 95% CI, 0.53–1.93) for SHS ≤14 h/wk and (OR = 1.91; 95% CI, 0.75–4.89) for SHS >14 h/wk in the models adjusted for tobacco use and several other potential confounding factors.

We found an indication of increased risk of ESCC associated with exposure to SHS. Studies with larger numbers of SHS-exposed never tobacco users are required to further examine this association.

## Abstract 2

Supplemental Digital Content is available in the text

## INTRODUCTION

Esophageal cancer is the eighth most commonly occurring cancer and the sixth leading cause of cancer deaths worldwide.^1^ Esophageal cancer has two main histological types, adenocarcinoma and squamous cell carcinoma.^2^ Esophageal squamous cell carcinoma (ESCC) is very common in certain regions in Asia, including Linxian of China,^3^ Golestan province of Iran,^4^ and Kashmir of India.^5^ The etiology of ESCC is complex and not completely understood yet. Previous studies in high-risk regions have shown several potential risk factors of ESCC, including low socioeconomic status (SES),^6–12^ poor oral hygiene,^13–17^ contact with animals,^18,19^ consumption of tea,^20,21^ and tobacco use in different forms.^22–26^ The share and contribution of tobacco smoking in ESCC development and mortality is likely to increase further in the developing countries as its consumption is worryingly increasing,^27,28^ and if such smoking patterns persist an epidemic of cancer attributed to tobacco smoke inhaled by active or secondhand smokers is expected to occur in developing countries.^29,30^

According to the International Agency for Research on Cancer, there is sufficient evidence for a causal association between secondhand smoking (SHS) and lung cancer, as well as limited evidence for the association with laryngeal and pharyngeal cancer.^31^ However, because SHS contains many carcinogenic compounds existing in the mainstream smoke, it may cause some other smoking-related cancers. The association between SHS and ESCC has not been investigated to the extent as studied with active smoking^32^ and the results of available studies are inconclusive. Two studies from China^3,33^ have reported positive associations, but none of those results were controlled for active smoking. A comparative study has reported no and positive association with ESCC in a high-risk and a low-risk region, respectively in China.^34^ Hence, it will be important to understand the role of SHS further in ESCC development in these high ESCC risk regions where the use of tobacco is on rise.

In Kashmir, ESCC is the most common cancer among both men and women.^5,35^ The joint family system is a characteristic feature of the Kashmiri society and *hookah* use, associated with ESCC risk,^26^ is a common practice in the presence of other family members. Therefore, the present study was conducted to assess the association between SHS and ESCC risk in Kashmir in both active tobacco users (smokers and chewers) and never tobacco users.

## MATERIALS AND METHODS

### Subject Selection

All cancer cases were recruited at the Regional Cancer Centre and Department of Radiation Oncology of Sher-i-Kashmir Institute of Medical Sciences (SKIMS) from September 2008 to January 2012. Every case in the study was histopathologically confirmed as ESCC and had no previous cancer. For each case, at least 1 control individually matched to the case for sex, age (±5 years), and district of residence was recruited from in-patient wards of SKIMS, the Government Medical College Hospital, Srinagar, and all 10 district hospitals of Kashmir. Informed and written consent was taken from all the subjects before recruitment. We tried to recruit >1 control for each case whenever possible. Most of the cases (54%) had 2 controls, whereas 44 cases (6%) had 1, 268 cases (38%) had 3, and 14 cases (2%) had >3 controls. Patients were enrolled as controls only when the disease for which they had been admitted did not have a strong association with tobacco or alcohol consumption. The participation rate for cases and control was 96% (732 invited, 30 refusals) and 98% (1697 invited, 34 refusals), respectively. The majority of those who refused were too ill to participate in the study. Other information about the study design and major reasons for hospitalizations of the enrolled controls are provided in detail elsewhere.^26^ This study was reviewed and approved by the Institutional Ethics Committee of SKIMS.

### Data Collection

Structured questionnaires were administered in face-to-face interviews at hospital by trained interviewers. A limited number of staff conducted the interviews and no proxies were used. Information was collected on demographic characteristics like age, sex, ethnicity, religion, place of residence, education, and several indicators of SES, including education level, ownership of several household appliances, house type, cooking fuel, and occupation. Dietary data, including intake of fresh fruits and vegetables, were collected using a food frequency questionnaire specifically designed for this population.

Detailed information was gathered on lifestyle habits, including smoking status, lifelong smoking history of *hookah*, cigarette, and *bidi*, ever use of alcohol and several other tobacco products such as *gutka* (a mixture of tobacco, areca nut, lime, and several other substances, such as flavorings and sweeteners) and *nass* (a mixture of tobacco, ash, lime, oil, and flavoring and coloring agents). Smokers of tobacco in any form (*hookah*, cigarette or *bidi*) were grouped as active smokers and active chewers include both *nass* and *gutka* users. The information was also collected about starting and stopping age and daily amount of use. The detailed information pertaining to the tobacco use is provided elsewhere.^26^

SHS is the inhalation of mixture of smoke from side-stream and exhaled mainstream tobacco smoke by others.^36,37^ Information on SHS exposure was obtained from the subjects regarding the number of active smokers who smoked in the participant's presence and the type of relation with the active smoker. The duration of secondhand smoke exposure was measured in hours and number of days per week spent by participant with active smoker. Almost all subjects reported their SHS exposure for whole week, which prompted us to classify the dose of SHS into ≤14 and >14 h / week’. Subjects with ≤2 h of exposure to secondhand smoke a day were categorized as “≤14 h exposure/wk” (≤2 h × 7 days = ≤14 h/wk) group and those with higher hours of exposure were included in “>14 h exposure/wk” category. These active smokers included the participant's spouse, parents, brothers, sisters, uncles, aunts, or other relatives in his/her home. The information was also collected on secondhand smoke exposure in workplaces and public settings.

### Statistical Analysis

Numbers and percentages were calculated and presented for categorical variables, as well as means and standard deviations (SD) or median and interquartile range for continuous variables. Fruit and vegetable intake data (g/d) were transformed to logarithmic values following addition of 0.1 to original values. To assess the SES, we built a composite score for wealth. The wealth scores were categorized as quintiles according to the observed coordinates among control subjects, the details of calculation are provided elsewhere.^6^

Conditional logistic regression was used to calculate unadjusted and adjusted odds ratios (ORs) and corresponding 95% confidence intervals (CIs). By design, case and control subjects were matched by age, sex, and district of residence. Adjusted ORs (95% CIs) were obtained from 2 models (Table 3). In the first model, OR_1_s (95% CIs) were adjusted for demographic factors, including age, ethnicity, religion, place of residence, income, sex, education, the wealth score, ever use of alcohol, frequency of close contact with animals, salt tea consumption, house type, cooking fuel, and fruit and vegetable intake, (logarithmic scale). In the second group of models OR_2_s (95% CIs), in addition to above demographic factors, potential confounding by active smoking was additionally adjusted by adding terms for cumulative use of cigarette, *hookah*, and *bidi* for active chewers and ever-use of *nass*, and *gutka* for active smokers. Age was included in the multivariate models, because the matching for age was not perfect (±5 years). We adjusted the results for religion because an earlier study from this region had suggested dissimilar incidence of ESCC among people with different religions.^38^ Although some people in Kashmir live in concrete houses, most of the people particularly in rural areas live in adobe houses. House type reflects SES in the Kashmiri population.^6^ In addition, to possible high SHS exposure, the other indoor exposures including smoke from using biomass as cooking fuel are expected to get aggravated in such houses due to poor ventilation. The quantum of smoke from biomass use as cooking fuel is much more than clean fuel. The people who lived in adobe houses mostly have low SES and likely use such fuels, which are affordable and readily available for them. Therefore, both house type and cooking fuel were used as controls in multivariate analysis. All statistical analysis was done using Stata software, version 12 (STATA Corp., College Station, TX). Two-sided *P* values < 0.05 were considered as statistically significant.

**TABLE 3:**
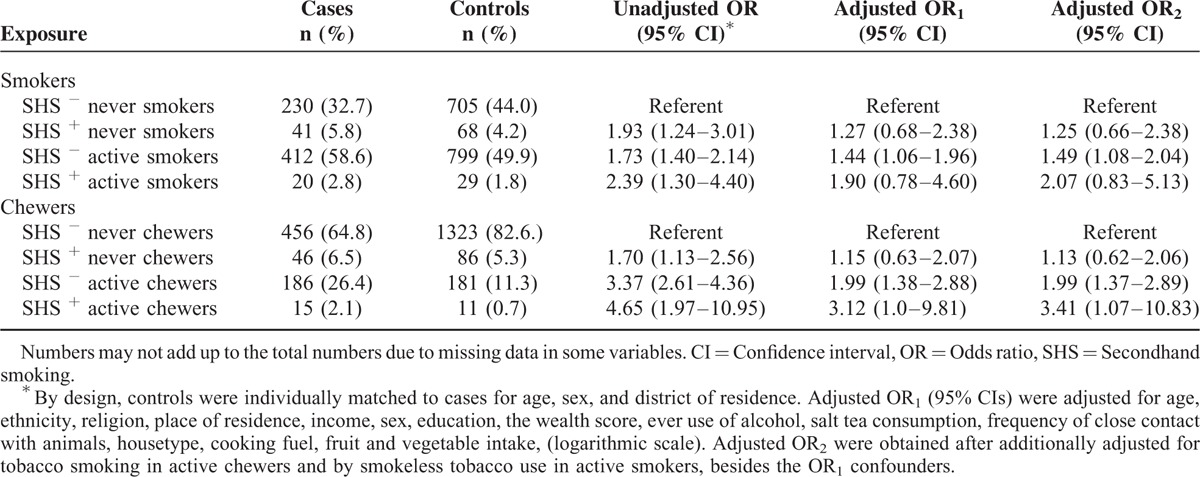
Association Between Exposure to Secondhand Smoke and the Risk of Esophageal Squamous Cell Carcinoma by Active Tobacco Smoking and Chewing Status, Kashmir, India

## RESULTS

A total of 703 ESCC cases and 1664 controls were enrolled in this study. The distribution of demographic variables in participants is shown in Table 1. The majority of study subjects were >50 years. Approximately 55% of cases were males and majority of cases were rural inhabitants and most of them lived in adobe houses. Formal education level and daily fruit and fresh vegetable intake were higher in controls than in ESCC cases. More than 50% of ESCC patients were active smokers and ∼30% of participants were active chewers.

**TABLE 1:**
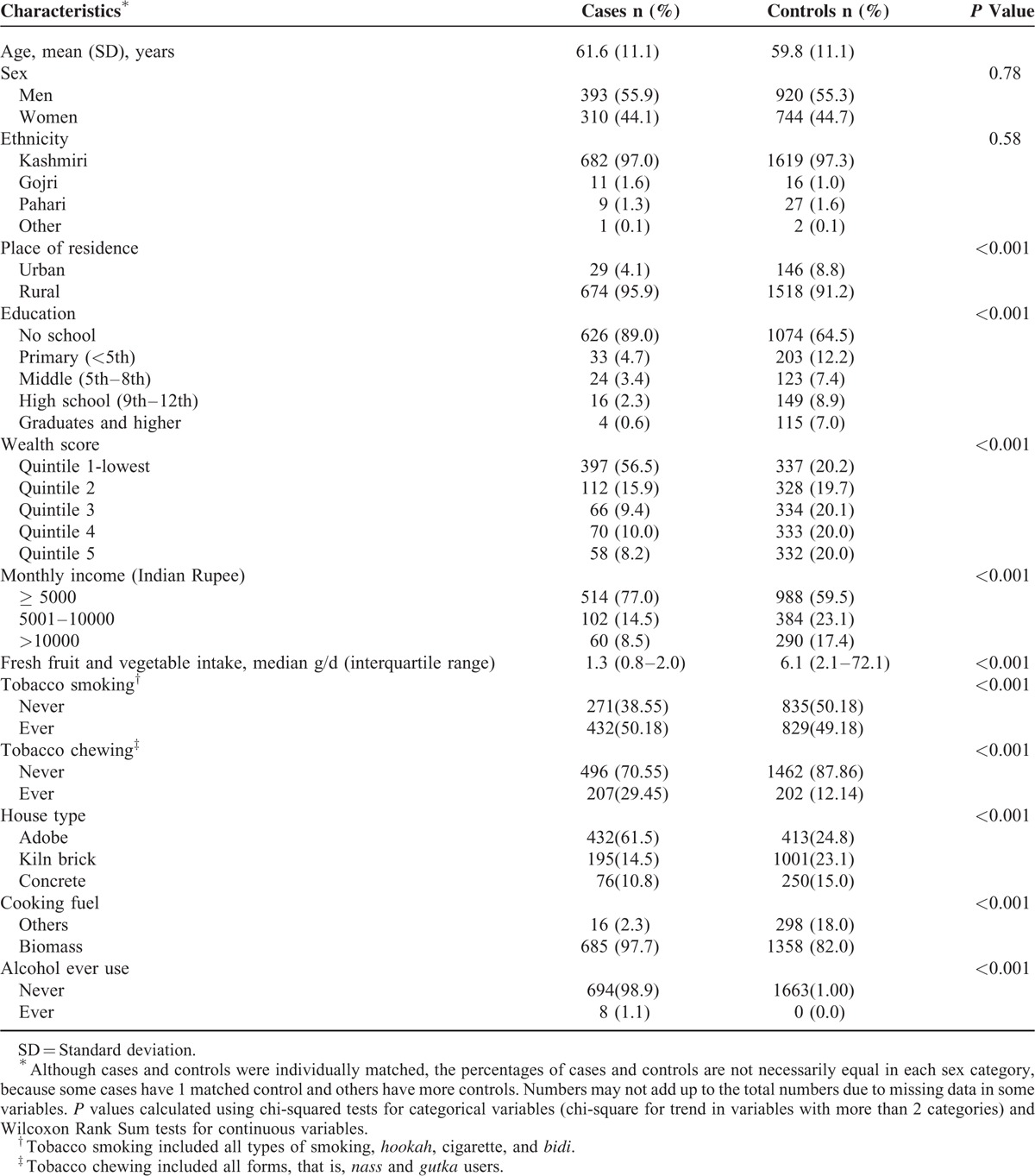
General Characteristics of 703 Esophageal Squamous Cell Carcinoma Cases and 1664 Matched Controls From Kashmir Valley, India

Table 2 shows the effects of SHS in secondhand smokers in general and in never tobacco users. Overall, SHS in the unadjusted model increased ESCC risk (OR = 1.64; 95% CI, 1.14–2.36); however, the association was attenuated and the 95% CI included unity (OR = 1.23; 95% CI, 0.72–2.11) in the models adjusted for tobacco smoking and chewing and other potential confounding factors. The OR (95% CI) for the association between weekly exposure to secondhand smoke for >14 h and ESCC risk, compared to no exposure, was (OR = 1.91; 95% CI, 0.75–4.89). When analysis was limited to never tobacco users (never smokers and never chewers) the OR (95% CI) for the association between SHS and ESCC risk, in adjusted model, was (OR = 1.32; 95% CI, 0.43–4.02) (Table 2). The OR increased with a higher exposure (OR = 2.69; 95% CI, 0.75–20.65) for SHS >14 h a week versus no exposure.

**TABLE 2:**
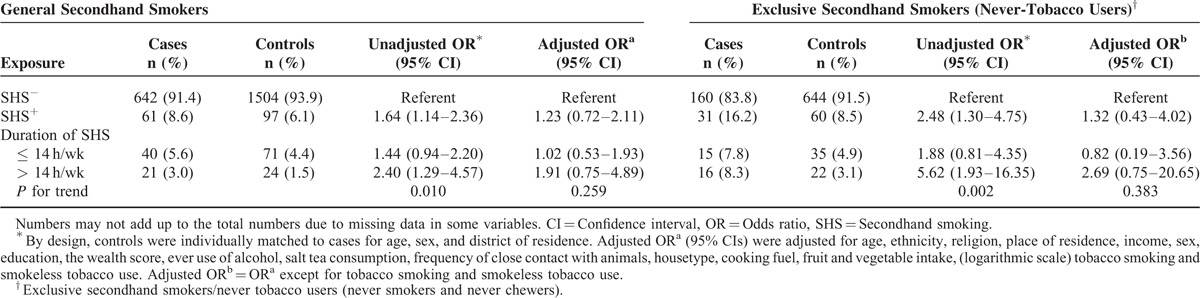
Association Between Exposure to Secondhand Smoke and the Risk of Esophageal Squamous Cell Carcinoma in General and Exclusive Secondhand Smokers (Never Tobacco Users) Kashmir, India

Table 3 shows the association of SHS with ESCC risk separately for tobacco smokers and chewers. On assessing the effects of the SHS in current smokers and chewers, significant increase in the ESCC risk was found. In adjusted models, there was no statistically significant difference in ESCC risk by SHS in active smokers. The OR was higher in tobacco chewers with SHS (OR = 3.41; 95% CI, 1.07–10.83) than tobacco chewers without SHS (OR = 1.99; 95% CI, 1.37–2.89), but there was no statistically significant interaction between SHS and tobacco chewing with regard to ESCC risk (Supplementary Table 1).

Further, in exclusive secondhand smokers or never tobacco users, we assessed the impact of important SES indicators, education and wealth score on association of SHS with ESCC risk. The secondhand smoke exposure was more common in participants who had no formal education or were poorer. In unadjusted models, we observed increased ESCC risk in individuals who had SHS exposure and without any formal education (OR = 2.95; 95% CI, 1.47–5.91) or who had lowest wealth score (quintile 1) (OR = 4.11; 95% CI, 1.50–11.2). However, after confounding with potential risk factors including several indicators of SES, such risk got disappeared in case of participants with no education but the association was reduced to border line significance in participants with lowest wealth score (Supplementary Table 2).

## DISCUSSION

We found an indication of increased risk of ESCC associated with exposure to SHS. Although the observed associations were not statically significant, our results suggest a dose-response borderline significant association for SHS >14 h per week. Further, the SHS exposure was more common in participants who lacked formal education or were poorer.

Tobacco smoking can explain the risk of ESCC in 90% of cases^39–41^ in the areas with a low incidence of ESCC; however, this proportion is much lower in high-incidence regions of ESCC, probably because intensity of smoking in low-incidence regions is not yet as high as in high-incidence areas.^41,42^ This relatively “low intensity” of active smoking might be one of the reasons, in addition to relatively modest number of people with SHS, for not observing statistically significant associations in this study.

Tobacco smoke contains mixture of harmful compounds and carcinogens that cause various cancers, including ESCC.^43,44^ Secondhand smoke is a mixture of smoke from sidestream and exhaled mainstream smoke, which is inhaled by secondhand smoker as well as by the active smoker itself.^32^ Mainstream smoke and sidestream smoke are known to contain largely the same carcinogenic components,^45^ including polynuclear aromatic hydrocarbons, tobacco-specific N-nitrosamines, volatile *N*-nitrosamines, tar, carbon monoxide, carbon dioxide, benzene, ammonia, nicotine, and benzo[*a*] pryene and aromatic amines (4-aminobiphenly). The harmful compounds in SHS^45–52^ are easily absorbed into blood and lymph than particulate phase constituents of mainstream smoke.^45^

The enhanced risk of ESCC in *nass* chewers with SHS can be linked to the additive effects of tobacco-related carcinogens in SHS and smokeless tobacco use. *Nass* chewing is a known risk factor for esophageal carcinogenesis in Kashmir^26^ and in other high-risk populations.^22,23,25^ The constituents of *nass*, such as polycyclic aromatic hydrocarbons from ash, may have carcinogenic effects on the esophageal epithelium.^53–55^

The joint family system and smoking in the presence of family members are common in Kashmiri population. People share the same living places especially kitchens where they sit together for hours. Hence, all the possible relatives including parents, siblings, and spouse, who smoke in the presence of participants, can be sources of SHS.

Majority of the Kashmiris are rural, adobe dwellers, with low SES.^6^ The SHS is more common in the low economic section of the Kashmiri society. The common SHS exposure in people with low SES can be attributed to many reasons. Although low SES is not a biological cause of cancer, it may influence the risk through behavior, lifestyle, environmental exposure, and diet. Education has been consistently used as a marker of SES and is inversely associated with risk of ESCC.^8,9^ Higher education may reflect higher SES of a family during childhood, which may have an effect on future health. In addition, people with higher education may be more likely to get well-paid job^56^ and obtain health-related knowledge, which may modulate cancer risk.^57^ In addition, wealth score based on ownership of some of the appliances may also be associated with lower risk in some other ways. For example, ownership of a TV may help people to obtain more health-enhancing information compared to those without a TV in their household.^6^ In other words, better economic status of a family may help in developing awareness and sensitivity about harmful effects of SHS.

Recruitment of histologically confirmed ESCC cases and individually matched controls from the same district of residence as cases, investigation of the association between SHS and ESCC risk in never tobacco users, and adjustment of the results for known potential confounding factors are among the strengths of this study. The major limitation of the study is the modest number of never tobacco users with a history of SHS, which was probably the main reason for which we did not find statistically significant associations in this group. Recall bias can be another limitation of this study because of its case-control setting. However, this bias is unlikely, because the majority of participants had little formal education and there was no earlier information on the association between SHS and risk of ESCC in this region.

## CONCLUSION

Our findings indicate increased risk of ESCC due to SHS exposure in dose-dependent manner. Our results may help to increase the awareness about harms of SHS, particularly in developing populations where tobacco use is on rise and ESCC incidence is high. However, more studies with a larger sample size are required before making any conclusion on the association between SHS and ESCC risk.

## Supplementary Material

Supplemental Digital Content
