# Supplementary material for: Secondhand Smoking and the Risk of Esophageal Squamous Cell Carcinoma in a High Incidence Region, Kashmir, India: A Case-control–observational Study
Source: Medicine (Baltimore). 2016 Jan 8;95(1):e2340. doi: 10.1097/MD.0000000000002340 (PMC4706255; doi:10.1097/MD.0000000000002340)
Supplement: Supplemental Digital Content [file medi-95-e2340-s001.docx]

**Supplementary Table 1: Interaction between active smoking and chewing with SHS**

| **Variable** | **Standard error** | **P-value** | **OR** | **95% CI** |
| --- | --- | --- | --- | --- |
| Active smoker*SHS ^X^ | 0.26 | 0.365 | 0.71 | 0.34 – 1.48 |
| Active chewer*SHS ^Y^ | 0.39 | 0.666 | 0.80 | 0.30 – 2.12 |

Abbreviations = OR, odds ratio; CI, confidence interval; SHS, Secondhand Smoking

^X^ Interaction between active smoking and SHS

^Y^ Interaction between active chewing and SHS

**Supplementary Table 2:** **Association between exposure to secondhand smoke and the risk of esophageal squamous cell carcinoma in exclusive secondhand smokers stratified by socioeconomic status indicators in Kashmir, India.**

| **Exposure** | **Cases**  **n (%)** | **Controls**  **n (%)** | **Unadjusted OR**  **(95% CI)*** | **Adjusted OR**  **(95% CI)** |
| --- | --- | --- | --- | --- |
| **Exclusive secondhand smokers ^#^** | | | | |
| **Education** |  |  |  |  |
| SHS ^-^ | 160 (83.7) | 644 (91.4) | Referent | Referent |
| SHS ^+^  Education ^+^ | 3 (1.6) | 20 (3.0) | 0.49 (0.05 – 4.80) | 1.20 (0.01 – 99.20) |
| SHS ^+^  Education ^-^ | 28 (15.0) | 40 (5.6) | 2.95 (1.47 – 5.91) | 1.39 (0.48 – 4.02) |
| **Wealth score** | | | | |
| SHS ^+^  Highest quintile | 4 (2.0) | 15 (2.1) | 1.15 (0.32 – 4.06) | 0.45 (0.05 – 3.95) |
| SHS ^+^ Intermediate quintile | 12 (6.3) | 27 (4.0) | 2.27 (0.88 – 5.80) | 0.83 (0.20 – 3.39) |
| SHS ^+^  Lowest quintile | 15 (8.0) | 18 (2.5) | 4.11 (1.50 – 11.2) | 6.34 (1.01 – 39.8) |

Abbreviations = OR, Odds ratio; CI, Confidence interval; SHS, Secondhand smoking.

Numbers may not add up to the total numbers due to missing data in some variables**.** *By design, controls were individually matched to cases for age, sex, and district of residence. Adjusted OR (95% CIs) were adjusted for age, ethnicity, religion, place of residence, income, sex, the wealth score, education, ever use of alcohol, salt tea consumption, frequency of close contact with animals, housetype, cooking fuel, fruit and vegetable intake, (logarithmic scale). However, the variable, when under consideration was not included in adjusted OR models. Education ^+^ = Primary to graduate or higher, Education ^- =^ No school, Highest quintile = Combination of quintile 4 and quintile 5, Intermediate quintile = Combination of quintile 2 and quintile 3, Lowest quintile = quintile 1. ^#^ Exclusive secondhand smokers / never tobacco users (never smokers and never chewers).
